# Optimization of liposomal topotecan for use in treating neuroblastoma

**DOI:** 10.1002/cam4.1083

**Published:** 2017-05-23

**Authors:** Lina Chernov, Rebecca J. Deyell, Malathi Anantha, Nancy Dos Santos, Roger Gilabert‐Oriol, Marcel B. Bally

**Affiliations:** ^1^ Experimental TherapeuticsBC Cancer Agency^th^675 West 10^th^ AvenueVancouverBritish ColumbiaV5Z 1L3Canada; ^2^ Department of Pathology and Laboratory MedicineUniversity of British Columbia2211 Wesbrook MallVancouverBritish ColumbiaV6T 2B5Canada; ^3^ Division of Pediatric Hematology/OncologyBritish Columbia Children's Hospital and the University of British Columbia4480 Oak StreetVancouverBritish ColumbiaV6H 3V4Canada; ^4^ Michael Cuccione Childhood Cancer Research ProgramBritish Columbia Children's Hospital Research Institute^th^950 West 28^th^ AvenueVancouverBritish ColumbiaV5Z 4H4Canada; ^5^ Faculty of Pharmaceutical SciencesUniversity of British Columbia2405 Wesbrook MallVancouverBritish ColumbiaV6T 1Z3Canada; ^6^ Centre for Drug Research and Development4‐2405 Wesbrook MallVancouverBritish ColumbiaV6T 1Z3Canada

**Keywords:** Camptothecin, lipids, liposomes, neuroblastoma, sphingomyelin, topotecan

## Abstract

The purpose of this work was to develop an optimized liposomal formulation of topotecan for use in the treatment of patients with neuroblastoma. Drug exposure time studies were used to determine that topotecan (Hycamtin) exhibited great cytotoxic activity against SK‐N‐SH, IMR‐32 and LAN‐1 neuroblastoma human cell lines. Sphingomyelin (SM)/cholesterol (Chol) and 1,2‐distearoyl‐*sn*‐glycero‐3‐phosphocholine (DSPC)/Chol liposomes were prepared using extrusion methods and then loaded with topotecan by pH gradient and copper‐drug complexation. In vitro studies showed that SM/Chol liposomes retained topotecan significantly better than DSPC/Chol liposomes. Decreasing the drug‐to‐lipid ratio engendered significant increases in drug retention. Dose‐range finding studies on NRG mice indicated that an optimized SM/Chol liposomal formulation of topotecan prepared with a final drug‐to‐lipid ratio of 0.025 (mol: mol) was better tolerated than the previously described DSPC/Chol topotecan formulation. Pharmacokinetic studies showed that the optimized SM/Chol liposomal topotecan exhibited a 10‐fold increase in plasma half‐life and a 1000‐fold increase in AUC_0–24 h_ when compared with Hycamtin administered at equivalent doses (5 mg/kg). In contrast to the great extension in exposure time, SM/Chol liposomal topotecan increased the life span of mice with established LAN‐1 neuroblastoma tumors only modestly in a subcutaneous and systemic model. The extension in exposure time may still not be sufficient and the formulation may require further optimization. In the future, liposomal topotecan will be assessed in combination with high‐dose radiotherapy such as ^131^I‐metaiodobenzylguanidine, and immunotherapy treatment modalities currently used in neuroblastoma therapy.

## Introduction

Neuroblastoma is the third leading cause of childhood cancer‐related mortality and is the most common and the deadliest pediatric extracranial solid tumor 1. It is a cancer of the sympathetic nervous system, and arises from the sympathoadrenal lineage of the neuronal crest 1, 2. With a median age at diagnosis of 17 months, most primary tumors arise within the adrenal medulla but may present along the paraspinal sympathetic ganglionic chain 1. Neuroblastoma is primarily sporadic with less than 2% of cases having a familial origin 1, 3. However, recent genome‐wide association studies suggest that common, low‐penetrance germline polymorphisms may predispose children to developing neuroblastoma, even in sporadic cases 4. Approximately 500–700 new patients are diagnosed every year in North America; more than 50% have a high‐risk disease who present with widespread metastases and have poor survival outcomes despite intensive, multi‐modality approaches to therapy.

Neuroblastoma is a very heterogeneous disease with a wide spectrum of clinical behaviors 1, 5. Patients younger than 18 months of age with limited metastasis (to liver, skin, <10% bone marrow involvement) and favorable tumor biology (absence of MYCN oncogene amplification and structural genetic abnormalities) have very good prognosis 1, 2, 6. However, treatment of older children presenting with unfavorable prognostic markers remains one of the greatest challenges for pediatric oncologists 7, 8. For high‐risk neuroblastoma patients, current standard treatment includes intensive, multi‐agent induction chemotherapy including topotecan, surgery, tandem high‐dose chemotherapy with autologous stem cell rescue and use of the differentiation agent 13‐cis retinoic acid, along with immunotherapy in maintanence 9, 10. Despite significant intensification of high‐risk therapy, there remains a large group of children with either primary refractory or relapsed disease and the 5 year event‐free survival (EFS) remains less than 50% 7, 10, 11.

Several targeted therapeutic approaches have a documented role in high‐risk neuroblastoma therapy. The incorporation of immunotherapy utilizing Dinutuximab, an anti‐GD2 monoclonal antibody, along with cytokines (GM‐CSF, IL‐2) in maintenance therapy has been shown to improve both EFS and overall survival (OS) in children with high‐risk neuroblastoma 12. An additional tumor‐specific agent used in neuroblastoma therapy is the targeted radiopharmaceutical ^131^I‐metaiodobenzylguanidine (^131^I‐MIBG) 10, 13. Treatments combining ^131^I‐MIBG with radiosensitizing agents such as Hycamtin (topotecan) or Camptosar (irinotecan) are now being investigated with the hopes of improving long term survival in patients with high‐risk neuroblastoma 10, 14, 15.

In addition to their radiosensitizing properties, camptothecins such as topotecan show significant clinical activity as single agents against neuroblastoma 16, 17, 18, 19. Topotecan is a water‐soluble analog of camptothecin which act to stabilize the covalent complex between topoisomerase I and DNA. This leads to irreversible double‐strand breaks and apoptotic cell death. Camptothecins are most lethal during the S‐phase of the cell cycle, therefore, prolonged drug exposure is important to maximize the cytotoxic activity of these agents 20, 21. Importantly, the therapeutic activity of topotecan depends on the chemical structure of the compound maintaining an intact lactone ring. The lactone moiety undergoes a pH‐dependent reversible hydrolysis into a nonactive carboxylate form at physiological pH (pH > 7). Thus, following intravenous administration, this drug rapidly (half‐life of <30 min) converts to the carboxylate form.

A variety of nanocarrier drug delivery systems have been used to protect camptothecins from this pH‐induced hydrolysis. More specifically, several liposomal formulations of camptothecins have been described 22, 23, 24, 25, and these are in addition to the two formulations developed in our laboratory: Irinophore C^™^ 26 and Topophore C^™^ 27. The former refers to an optimized liposomal formulation of irinotecan while the latter is a topotecan formulation prepared using the same methods described for Irinophore C^™^. Topophore C^™^, consisting of DSPC/Chol at a final drug‐to‐lipid ratio of 0.1 (mol: mol), exhibited significant antitumor activity in models of ovarian cancer, however, it was significantly more toxic than the clinical formulation (Hycamtin) and it released associated topotecan rapidly, with more than 98% drug loss within 8 h following intravenous administration 27.

The goal of this study was to optimize a liposomal formulation of topotecan as a candidate product for the treatment of neuroblastoma. We explored strategies to obtain formulations that exhibit improved drug retention based on the belief that enhanced drug retention should result in enhanced therapy. By selective changes in liposomal lipid composition and controlling the drug‐to‐lipid ratio, the resultant formulation increased the plasma circulation half‐life and AUC_0–24_ of the associated topotecan when compared to the free drug over 24 h. The amount of drug reaching the tumor site increased 25‐fold when compared to the clinical product administered at the same dose. The novelty of the manuscript lies on the fact that it is the first report assessing the therapeutic activity of copper based liposomal topotecan in models of neuroblastoma (both subcutaneous and systemic mouse models). The optimized formulation showed activity superior to the free drug as measured by delay in tumor growth and increase in median survival.

## Materials and Methods

### Materials and chemicals

1,2‐Distearoyl‐*sn*‐glycero‐3‐phosphocholine (DSPC), N‐hexadecanoyl‐D‐*erythro*‐sphingosyl phosphorylcholine (sphingomyelin, SM) and cholesterol (Chol) were obtained from Avanti Polar Lipids, Inc. (Alabaster, AL). ^3^H‐cholesteryl hexadecyl ether (^3^H‐CHE) was purchased from PerkinElmer Inc. (Waltham, MA). Pico‐Fluor 40 scintillation cocktail was obtained from PerkinElmer Life Sciences (Woodbridge, ON, Canada). Hycamtin injection (topotecan) was purchased from BC Cancer Agency (Vancouver, BC, Canada). Methanol, HPLC Grade was obtained from Alfa Aesar (Massachusetts). Glacial acetic acid, Sucrose, HEPES, Sephadex G‐50, EDTA, A23187 and all other chemicals came from Sigma‐Aldrich Canada Co. (Oakville, ON, Canada).

### Cell culture

IMR‐32 and SK‐N‐SH human neuroblastoma cells came from the American Type Culture Collection (ATCC) (Manassas, VA) and LAN‐1 human neuroblastoma cells came from DSMZ (Braunschweig, Germany). IMR‐32 and SK‐N‐SH were maintained in EMEM (Life Technologies, Carlsbad, CA) supplemented with 10% fetal bovine serum (FBS)/2 mmol/L l‐glutamine (Life Technologies). LAN‐1 were maintained in RPMI 1640 (Life Technologies) supplemented with 20% heat‐inactivated FBS/2 mmol/L l‐glutamine. Cells were passaged for no more than 10 times before returning to original stock cells from the supplied sources specified above.

### Cell viability assay

The cytotoxic activity of topotecan (Hycamtin) against SK‐N‐SH, IMR‐32 and LAN‐1 neuroblastoma cell lines was measured by assessing changes in cell viability as determined by the PrestoBlue^®^ Cell Viability Reagent (ThermoFisher Scientific, Burlington, ON, Canada). The concentration of drug that decreased the viability of cells by 50% defined the IC_50_ of the drug. To evaluate the effect of exposure time, SK‐N‐SH, IMR‐32 and LAN‐1 neuroblastoma cell lines were incubated with increasing concentrations of the drug for 1, 4, 8, 24, 48, or 72 h. Following each time point, the drug containing medium was removed and replaced with 200 *μ*L of fresh medium and the cells were incubated such that all cells were maintained in culture for a total of 72 h. The cell viability was then determined by measuring fluorescence (excitation at 544 nm and emission at 590 nm) with a FLUOstar OPTIMA Spectrophotometer (BMG Labtechnologies).

### Liposomes preparation

Liposomes were prepared using an extrusion method described by Hope et al. 28. Briefly, the lipids (DSPC/Chol or SM/Chol; 55:45 mol: mol) were dissolved in ethanol (100 mg lipid/mL) with ^3^H‐CHE, a nonexchangeable, nonmetabolizable radiolabeled lipid marker. The lipid mixture was then mixed with 300 mmol/L CuSO_4_ solution preheated to 60°C (1 mL ethanol/5.66 mL CuSO_4_ solution); resulting in a final ethanol concentration of 15% (v/v). The lipid structures were then processed using the extrusion method with two stacked polycarbonate filters of 0.1 and 0.08 *μ*m pore size at 60°C (Extruder^™^, Northern Lipids, Vancouver, BC). The size of the resultant liposomes was assessed using Phase Analysis Light Scattering (ZetaPALS, Brookhaven Instruments Corp., Holtsville, NY) which indicated that the mean diameter of the liposomes was 100 ± 20 nm. Tangential flow diafiltration (Watson Marlow 232 Pump, Falmouth, UK) was used to remove remaining ethanol. The resulting liposomal solution was then passed through a Sephadex G‐50 column equilibrated with SHE buffer (300 mmol/L Sucrose, 20 mmol/L HEPES and 15 mmol/L EDTA, pH 7.5) to remove unencapsulated copper. Liposomal lipid concentration was determined by measuring ^3^H‐CHE using liquid scintillation counting (LSC), with addition of 5 mL of Pico‐Fluor 40 scintillation cocktail. Topotecan was encapsulated in liposomes using the same encapsulation method developed for Irinophore C^™^ and Topophore C^™^; a method that relied on pH gradient and copper‐drug complexation 26, 27. This is described in detail below.

### Measurement of copper concentration

Copper concentration was determined using atomic absorption spectrometry (AAS) (AANALYST 600 PerkinElmer Instruments, Woodbridge, ON, Canada). A hollow cathode lamp (Cu‐LUMINA.HCL) was used as a light source for copper detection. Liposomal samples were diluted in nitric acid to achieve a final nitric acid concentration of 0.1%. Diluted samples were injected into the analysis chamber of the AAS and absorbance was measured at 325 nm. Copper concentration was determined against a freshly prepared standard curve (copper concentration range from 25 ng/mL to 100 ng/mL).

### Cryo‐electron microscopy (CEM)

CEM analysis was performed using a Zeiss Libra 120 transmission electron microscope at the University of Uppsala, Sweden. Briefly, topotecan loaded liposomes were prepared as described above. In a controlled chamber for humidity and temperature (25°C), 1–2 *μ*L sample was deposited on copper grids coated with a holey cellulose acetate butyrate polymer. Excess liquid was blotted away carefully with filter paper and then samples were quickly vitrified by plunging into liquid ethane. This was then transferred to liquid nitrogen to maintain the temperature and minimize formation of ice crystals. Images were taken in a zero‐loss bright‐field mode and an accelerating voltage = 80 kV.

### Analytical methods for quantification of topotecan

The drug was quantified using methods previously established in our laboratory 27, 29. For in vitro studies drug concentration was determined by diluting samples (90% v/v) in acidified methanol (3% v/v acetic acid, 97% v/v methanol). Subsequently, the absorbance was measured at 383 nm (Agilent/Hewlett Packard UV‐spectrophotometer, model: 8453, Agilent Technologies, Mississauga, ON, Canada). For in vivo derived samples or samples containing >10% serum protein, drug levels were assessed by a high‐performance liquid chromatography (HPLC) method using Waters Alliance HPLC system equipped with a Model 2474 Multi *λ* Fluorescence Detector (Waters, Milford, MA) set at an excitation wavelength of 380 nm and an emission wavelength of 525 nm. Samples were mixed with 3% acidified methanol, centrifuged at 14,000 rpm for 10 min to remove precipitated plasma proteins, and 10 *μ*L of an appropriately diluted supernatant was injected into a Water Symmetry Shield RP C_18_ column (5 *μ*m, 100 Å, 4.6 × 100 mm) adjusted to 55°C. The samples were maintained at 4°C before injection. Each sample was run for 10 min at flow rate of 1 mL/min, using mobile phase consisting of 30% solvent A (100% methanol) and 70% solvent B (1% TEA in water with the pH adjusted to 6.4 with acetic acid). Topotecan drug levels in tissues were analyzed by homogenizing the organ in cold PBS and mixing the homogenate with cold 3% acidified methanol before centrifugation at 14,000 rpm for 10 min. The resultant supernatant was processed for analysis by HPLC as described above.

### Topotecan encapsulation and release assays

Topotecan was encapsulated into liposomes prepared in 300 mmol/L CuSO_4_ (pH 3.5) and subsequently processed such that the external buffer was SHE buffer (300 mmol/L Sucrose, 20 mmol/L HEPES and 15 mmol/L EDTA, pH 7.5). The transmembrane pH gradient was maintained by the addition of A23187 (0.5 *μ*g per 1 mg of lipid). The mixture was incubated at 60°C for 15 min before the addition of topotecan. Immediately after the addition of topotecan the pH was adjusted to pH 7.5 with 1 N NaOH. This mixture was incubated at 50°C for 30 min, and then unencapsulated topotecan was removed by tangential flow dialysis against PBS (pH 7.5). For studies measuring topotecan loading efficiency, at the indicated time point samples were passed through 1 mL Sephadex G‐50 spin columns equilibrated with PBS. Topotecan in the eluent (liposome‐associated drug) was measured as described above. Liposomal lipid in the eluent was estimated by measuring ^3^H‐CHE by liquid scintillation counting (LSC).

For release assays, liposomes were diluted to a final topotecan concentration of 1.09 *μ*mol/mL with PBS (pH 7.5) and then 200 *μ*L of this sample was mixed with 1 mL FBS. The resulting mixture was incubated at 37°C and at the indicated time points, 100 *μ*L aliquots were fractioned on 1 mL Sephadex G‐50 spin columns.

### Pharmacokinetic and biodistribution studies

The selected formulation (SM/Chol liposomal topotecan with a drug‐to‐lipid mole ratio of 0.025) was diluted to the appropriate concentration in PBS (pH 7.5) such that the specified dose (5 mg/kg) could be administered in a volume of 10 *μ*L/g body weight. NRG male mice (6–8 weeks) were injected subcutaneously (right ventral flank region) with LAN‐1 cells mixed in matrigel (2.5 × 10^6^ cells per animal). When the tumor size was approximately 100–200 mg, the animals were randomized and treatment (intravenously (i.v.), dose of 5 mg/kg) was started on the following day. Tumor size was measured using a caliper and the measured dimensions (mm) were converted to tumor weight (mg) using the equation length × (width^2^) ÷ 2. At selected time points of the treatment, the animals were euthanized by isoflurane followed by CO_2_ exposure and blood was collected by cardiac puncture and placed into EDTA containing microtainers. Plasma was separated by centrifuging samples at 2500 rpm for 15 min at 5°C. The concentration of topotecan and liposomal lipid in the plasma samples were determined as described above. The plasma AUC (area‐under‐the‐curve) and half‐life of topotecan were determined using PK Solution 2.0, Noncompartmental Pharmacokinetics Data Analysis software. Harvested tissues were placed into preweight containers, weighed and frozen until analyzed for drug and liposomal lipid. A portion of the homogenized tissue was processed for measuring topotecan levels, and another portion was prepared for measuring ^3^H‐CHE. The topotecan levels were determined as described above. To measure ^3^H‐CHE, 200 *μ*L of tissue homogenates were mixed with 500 *μ*L of Solvable^™^ (PerkinElmer) and then heated at 50°C overnight before addition of 50 *μ*L 200 mmol/L EDTA and 200 *μ*L 30% H_2_O_2_. Five mL of Pico‐Fluor 40 scintillation cocktail was added, and ^3^H‐CHE was measured using LSC.

### Evaluation of toxicity of free and SM/Chol liposomal topotecan

Dose range finding studies were used to define tolerability of the formulation selected for further in vivo studies. Tumor free NRG mice (6–8 weeks) were injected i.v. using a Q7D × 3 schedule (2.5, 5, 7.5 or 10 mg/kg SM/Chol liposomal topotecan and 10 mg/kg Hycamtin as comparison) and the health status of the animals was monitored following an established standard operating procedure. In particular, signs of ill health were based on body weight loss, change in appetite, and behavioral changes such as altered gait, lethargy and gross manifestations of stress. The staff recording signs of toxicity were blinded to the treatment groups. When signs of severe toxicity were present, the animals were terminated (isoflurane overdose followed by CO_2_ asphyxiation) for humane reasons. Necropsy was performed to assess other signs of toxicity. The animals were monitored for 2 weeks (14 days) after administration of the last dose and full necropsies were completed on all treated mice.

### Antitumor activity in murine models of LAN‐1 neuroblastoma

Antitumor activity of SM/Chol liposomal topotecan with a drug‐to‐lipid mole ratio of 0.025 was evaluated in a subcutaneous (s.c.) and systemic (intra‐cardiac) model of neuroblastoma established in NRG male mice (6–8 weeks). The s.c. model (8 mice/group) was established as described before and treatment (5 mg/kg SM/Chol liposomal topotecan or 10 mg/kg Hycamtin, Q7D × 3) was initiated when the tumor size was between 50 and 150 mg. Animals were randomised just before starting the treatment and assessed contemporaneously. Tumor size was measured every other day until the estimated tumor mass exceeded 800 mm^3^ (the defined humane endpoint) or when the tumor ulcerated.

Systemic model was achieved by intra‐cardiac (i.c.) injection of LAN‐1 cells. Animals (8 mice/group) were anesthetized using isoflurane. 1 mL‐Syringe attached to a 26G needle was inserted at a 30 degree angle, immediately caudal to the xyphiod process, aiming toward the left shoulder of the animal. LAN‐1 cells (1.5 × 10^6^) were injected slowly in a volume of 100 *μ*L. Fourteen days after cell injection, animals were randomized and contemporaneously given the specified formulation i.v. (Q7D × 3 schedule) at the indicated drug dose. Health status of the animals was monitored at least twice per day in a daily basis for signs of morbidity due to treatment and/or tumor progression. When an animal reached a defined humane endpoint it was terminated (isoflurane followed by CO_2_ asphyxiation) and a necropsy was performed. The time of death was recorded as the following day. All animal studies were completed under an animal care protocol reviewed and approved by the University of British Columbia's Animal Care Committee (ACC). The studies met current guidelines of the Canadian Council of Animal Care. Animal studies were conducted only once due to ethical considerations.

### Statistical analysis

All statistical data was collected using GraphPad Prism (San Diego, CA). The log‐rank test was used to compare the survival curves of various treatment groups against appropriate controls. Differences between two groups were considered significant if *P *<* *0.05.

## Results

### Topotecan is a potent drug when used against neuroblastoma cell lines

**Figure 1:**
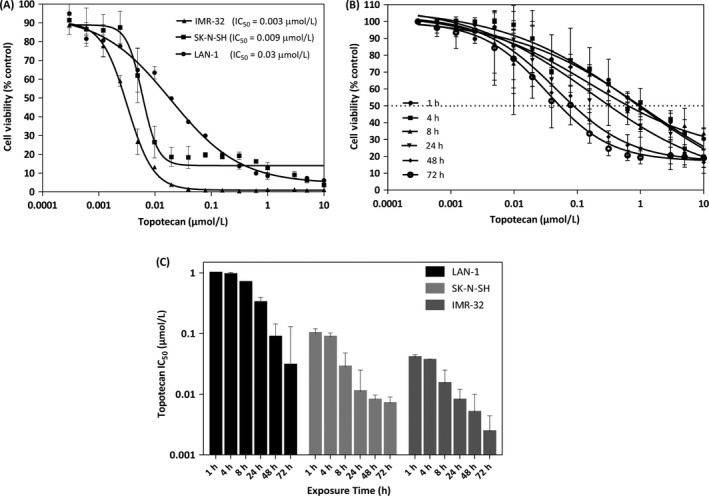
Topotecan is a potent drug when used against IMR‐3, SK‐N‐SH and LAN‐1 neuroblastoma cell lines. Dose‐response curves for topotecan (panel A) were determined in neuroblastoma cell lines using PrestoBlue^®^ as described in the Methods. The IC_50_ for the drugs are indicated. Changes in LAN‐1 cell viablity as a function of different topotecan exposure times (panel B) were determined. The IC_50_ as a function of topotecan exposure time for each cell line are summarized in panel C. Results are compared to untreated controls and presented as the mean ± SD of at least three independent experiments performed in triplicate.

The cytotoxic activity of topotecan was assessed in three neuroblastoma cell lines: IMR‐32, SK‐N‐SH and LAN‐1 cells and the results are summarized in Figure 1A. The IC_50_ values reported here for topotecan (3 to 30 nmol/L) are consistent with those previously reported (0.71–489 nmol/L) 30. The activity of topotecan is highly dependent on exposure time and this is illustrated by the data summarized in Figure 1B and 1C. For all three neuroblastoma cell lines evaluated, extended exposure to topotecan resulted in significant decreases in IC_50_. Specifically, for LAN‐1 cells as the exposure time was increased from 4 h to 72 h the IC_50_ of topotecan decreased 30‐fold from almost 1 *μ*mol/L (4 h exposure) to less than 0.03 *μ*mol/L (72 h exposure). Similar decreases in IC_50_ were observed for SK‐N‐SH and IMR‐32 neuroblastoma cells. A primary justification for formulating topotecan into nanoscaled drug delivery systems is the potential that increased topotecan exposure time, engendered by the drug delivery system, should result in enhanced efficacy in vivo.

### Optimization of a liposomal topotecan formulation

**Figure 2:**
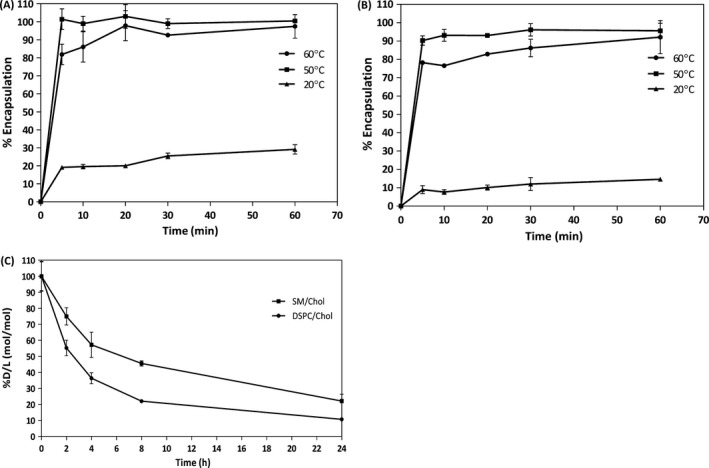
The SM/Chol liposomal topotecan formulation retains topotecan better than DSPC/Chol liposomal topotecan. The liposomes were prepared with unbuffered 300 mmol/L copper sulfate (pH3.5) and the divalent cation ionophore A23187 was added to help maintain the pH gradient following addition of topotecan (0.1 mol topotecan per mole liposomal lipid). Following drug addition, the pH of the solution was immediately adjusted to 7.5. The amount of liposome associated topotecan was determined at the indicated time points as described in the Methods. The results for SM/Chol (panel A) and DSPC/Chol (panel B) liposomes represent the mean ± SD for experiments repeated at least three times. In vitro topotecan release from SM/Chol and DSPC/Chol liposomes (loaded with topotecan using an incubation temperature of 50°C for 30 min) was determined following incubation of the indicated formulation in 80% FBS at 37°C. The amount of retained topotecan (% of initial drug (D) to lipid (L) ratio) was determined over a 24 h time course. Data points represent the mean ± SD for experiments done in duplicate and repeated three times (panel C).

Topotecan loading efficiency for the SM/Chol (Fig. 2A) and DSPC/Chol (Fig. 2B) topotecan formulations was the highest at the loading temperatures of 50 and 60°C after 60 min of topotecan addition. Stability of these formulations was assessed in vitro*,* by comparing drug release rate in the presence of FBS (80%) over an incubation time period of 24 h. The results, summarized in Fig. 2C, indicate that topotecan retention was significantly better for the SM/Chol liposomes when compared to the DSPC/Chol liposomes. The time required to release 50% of the encapsulated drug increased almost threefold for the SM/Chol formulation when compared to the DSPC/Chol formulation. After 8 h at 37°C, the SM/Chol formulation retained more than twice the amount of topotecan when compared to the DSPC/Chol liposomes.

Previous studies suggested that drug retention can increase in formulations exhibiting higher drug‐to‐lipid ratios; an effect thought to be due to drug precipitation within the liposome core 31. For the loading method described here, however, it can be suggested that faster drug release rates would be associated with loss of the transmembrane pH gradient 32 as well as loss of encapsulated copper which is known to complex topotecan and affect drug retention. Consequently, it can be suggested that increased internal copper levels and minimal dissipation of the pH gradient could result in improved drug retention. To test this SM/Chol liposomes were prepared at a 0.1 and 0.025 drug‐to‐lipid ratio (mol: mol) and the rate of topotecan dissociation from the liposomes was determined.

**Figure 3:**
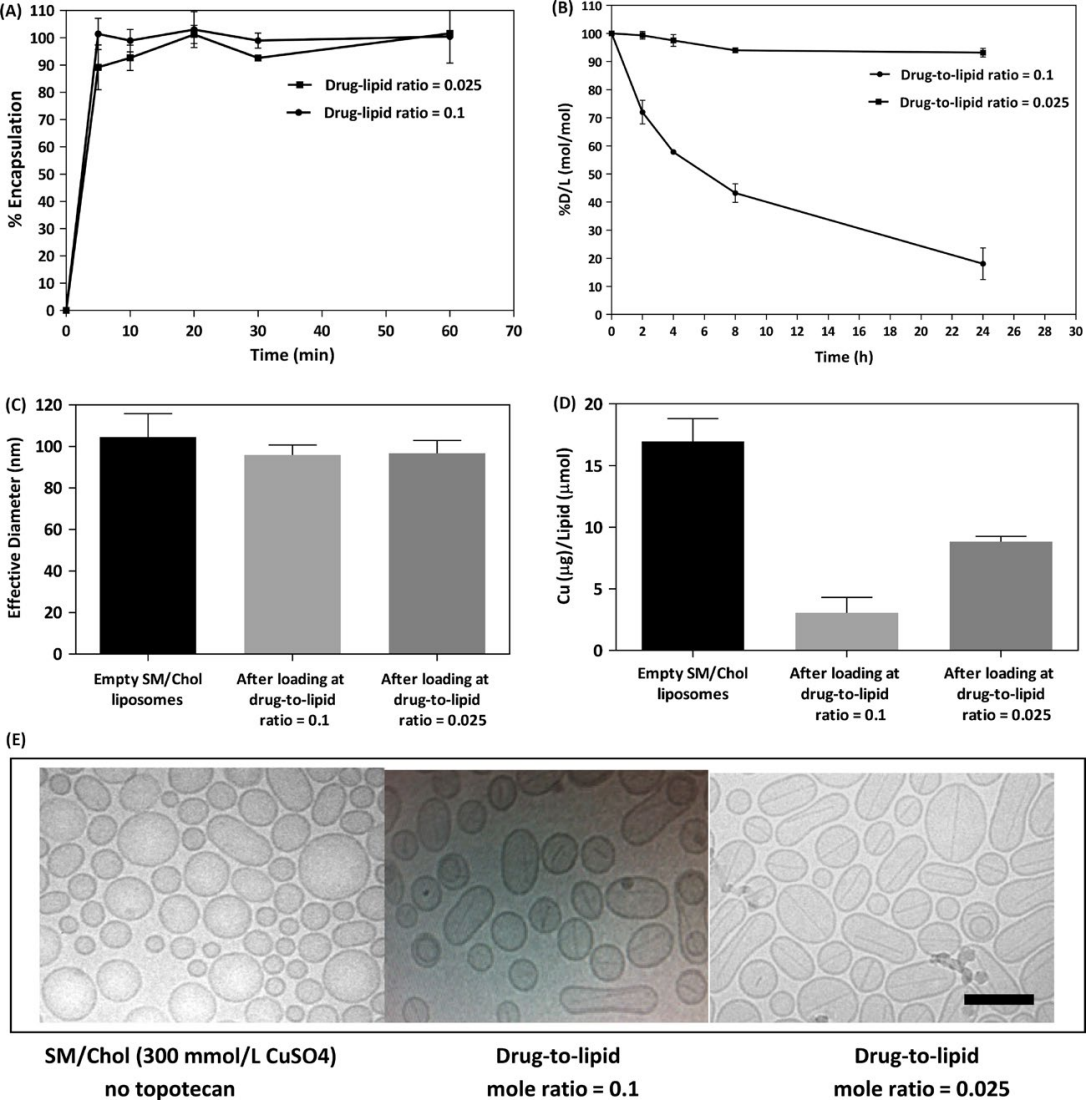
Liposomal topotecan formulations prepared at a 0.025 drug‐to‐lipid mole ratio retained drug better than liposomes prepared at a 0.1 drug‐to‐lipid mole ratio. Topotecan was loaded into SM/Chol liposomes at the indicated drug‐to‐lipid ratio using an incubation temperature of 50°C (panel A). The effect of drug‐to‐lipid ratio on topotecan release from SM/Chol liposomal formulations following incubation in 80% FBS at 37°C over 24 h is shown in Panel B. Decreases in the drug‐to‐lipid ratio represents loss of topotecan from the liposmes over time and each data point represent the mean ± SD of experiments repeated at least three times. The size of SM/Chol liposomes with and without encapsulated topotecan (drug‐to‐lipid mole ratios of 0.1 and 0.025) is shown in panel C; where size was determined using Phase Analysis Light Scattering. The amount of liposome associated copper (*μ*g copper/*μ*mol lipid) before and after topotecan encapsulation (drug‐to‐lipid mole ratios of 0.1 and 0.025) is shown in Panel D, where copper was measured using AAS. Representative cryo‐electron microscopy (CEM) images of SM/Chol liposomes before and after topotecan encapsulation (drug‐to‐lipid mole ratios of 0.1 and 0.025) are shown in panel E. The scale bar is 100 nm.

The results, summarized in Figure 3, indicate that the drug loading rate at 50°C was comparable for both drug‐to‐lipid ratios (Fig. 3A), however, the rate of drug dissociation at 37°C in the presence of 80% FBS was significantly slower for the formulation prepared at the lower drug‐to‐lipid ratio. There was <10% loss of encapsulated topotecan over 24 h under these in vitro conditions for the 0.025 drug‐to‐lipid ratio formulation as compared to >80% loss for the formulations prepared at 0.1 drug‐to‐lipid ratio. The two liposomal formulations exhibited the same size as determined by Phase Analysis Light Scattering (Fig. 3C). The amount of retained copper was reduced sevenfold for the formulations prepared at the 0.1 drug‐to‐lipid ratio whereas for the 0.025 drug‐to‐lipid formulation it was only about twofold lower (Fig. 3D). For the 0.025 drug‐to‐lipid formulation it can be estimated that the copper to topotecan molar ratio is 5. In contrast the copper to topotecan ratio in the 0.1 drug‐to‐lipid formulation is 10‐fold lower.

Cryo‐electron microscopy (CEM) was used to assess liposome structure and these studies revealed that the liposomal formulations of topotecan, regardless of final drug‐to‐lipid ratio, exhibited a fine needle‐like electron dense structure within the liposomes. As suggested by the representative micrographs shown in Figure 3E, the SM/Chol liposome without encapsulated topotecan appeared more spherical than formulations containing topotecan. The presence of the electron dense needle‐like crystal is comparable to what was reported previously for the DSPC/Chol topotecan formulations 33.

### In vivo characterization of the SM/Chol 0.025 topotecan‐to‐lipid mole ratio formulation

**Figure 4:**
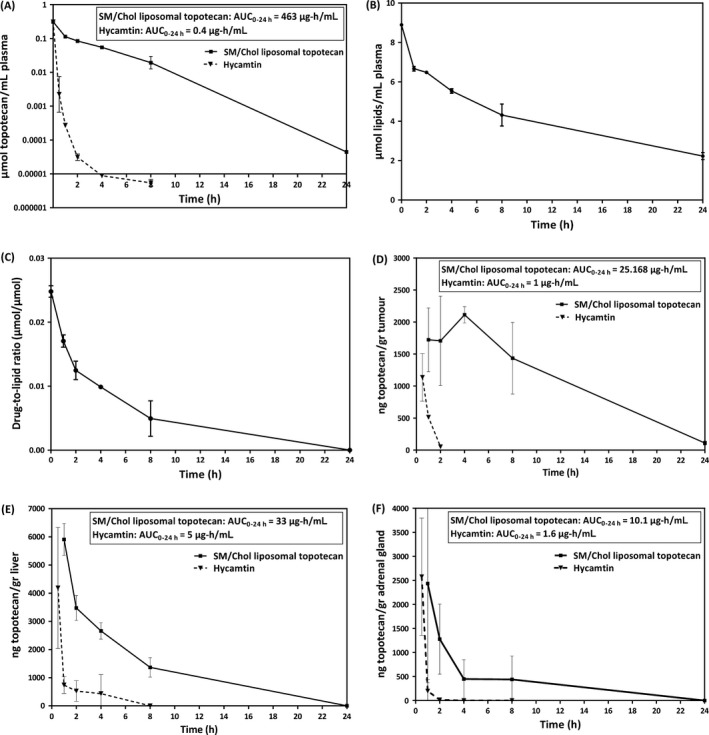
Administration of SM/Chol liposomal topotecan, in comparison to Hycamtin, enhances drug exposure in the plasma compartment, tumor, liver and adrenal gland. Pharmacokinetics and biodistribution were assessed after a single 5 mg/kg dose of topotecan administered as the SM/Chol liposomal formulation or Hycamtin. Formulations were administered i.v. into NRG mice with established s.c. LAN‐1 tumors. Plasma topotecan levels following administration of Hycamtin (panel A, filled triangles) and SM/Chol liposomal topotecan (panel A, filled squares) were determined by HPLC analysis as described in the Methods. Plasma liposomal lipid levels following administration of SM/Chol liposomal topotecan is shown in panel B, where liposomal lipid was measured using ^3^H‐CHE as a liposomal lipid marker by liquid scintillation counting (LSC). The calculated change in drug‐to‐lipid ratio in the plasma compartment following administration of SM/Chol liposomal topotecan is shown in panel C. Topotecan levels in tumor, liver and adrenal gland after administration of Hycamtin or the SM/Chol liposomal topotecan formulation are shown in panel D, E and F, respectively. Data points represent mean ± SEM obtained using at least three animals per group.

Pharmacokinetic and limited biodistribution data for Hycamtin (the clinical product) and SM/Chol liposomal topotecan (0.025 drug‐to‐lipid mole ratio) given as single i.v. dose of 5 mg/kg have been summarized in Figure 4. This study was conducted in NRG mice bearing established s.c. LAN‐1 neuroblastoma tumors. Following injection of Hycamtin, >99% of the injected drug was eliminated from the plasma compartment within 1 h (Fig. 4A). Topotecan concentration in the plasma was <0.04 *μ*mol/L 2 h after administration and at time points beyond 4 h topotecan levels were below the detection limits of the assay. In contrast, topotecan levels in the plasma compartment following administration of the SM/Chol liposomal topotecan formulation were detectible over the full 24 h time course. The difference between the formulations is emphasized by differences in plasma AUC_0–24 h_ for Hycamtin (0.4 *μ*g * h/mL) and SM/Chol liposomal topotecan (463 *μ*g * h/mL); where there was an increase in AUC_0–24 h_ of >1000‐fold (Fig. 4A insert). Liposomal lipid elimination following injection of the SM/Chol liposomal topotecan formulation (Fig. 4B) indicates that greater than 20% of the injected liposomal lipid dose was still in the plasma compartment at 24 h. Since greater than 99% of the topotecan was eliminated at this time point, these data indicate that the vast amount of liposome associate topotecan was released from the liposomes over 24 h. This is illustrated by the calculated drug‐to‐lipid ratio data summarized in Figure 4C. This data suggests that approximately 90% of the associated drug is released from the SM/Chol liposomes within 8 h. The level of topotecan measured at 8 h represents a 10‐fold improvement over the previously described DSPC/Chol formulation 27.

Following administration of SM/Chol liposomal topotecan there was also a significant increase in topotecan accumulation in the s.c. LAN‐1 tumors when compared to animals given Hycamtin (Fig. 4D). After 2 h of administration of Hycamtin, topotecan levels in the tumor were just at the detection limits of the assay while at the same time point topotecan levels were more than 100‐fold higher in animals given the SM/Chol liposomal topotecan formulation. In this case, there was a 25‐fold increase in tumor AUC_0–24 h_ when using the liposomal formulation. Figure 4E and 4F summarize data that compares levels of topotecan in the liver and adrenal gland, tissues known to be common sites of neuroblastoma growth. In both tissues there were significant increases in topotecan levels over time following administration of SM/Chol liposomal topotecan compared to Hycamtin, however, the fold increase in AUC_0–24 h_ (~6 to 7‐fold for both tissues) was much less than that seen in the tumor tissue or blood. While accumulation of topotecan in the liver is positive in view of treatment for possible metastasis, it is worth mentioning that this could be also associated with increased liver toxicities and a closer examination should be placed on this when considering dose augmentations.

### Antitumor activity of SM/Chol liposomal topotecan and Hycamtin in animals with established s.c. and systemic LAN‐1 neuroblastoma

Prior to initiating efficacy studies, dose range finding studies in tumor‐free NRG mice were completed to establish tolerability. The results have been summarized in Table S1. At 10 mg/kg, the SM/Chol topotecan formulation caused significant weight loss (~12%) following the first treatment. The mice recovered within 6 days and no further signs of treatment related morbidity were noted. The maximum feasible dose of Hycamtin was 10 mg/kg and it appeared to be better tolerated than the liposomal formulation as judged by body weight loss. However, there were signs of toxicity comparable to that noted in animals given SM/Chol liposomal at the 7.5 and 10 mg/kg dose. The frequency of effects was greater in animals treated with 7.5 mg/kg (4/4 mice effected) when compared to those animals treated with 10 mg/kg Hycamtin. Based on these data, the equitoxic doses were defined as 10 mg/kg and 7.5 mg/kg for Hycamtin and SM/Chol liposomal topotecan, respectively.

Results from the development studies of LAN‐1 neuroblastoma model are summarized in Figure S1. This includes survival curves for untreated animals and representative micrographs of tumor tissue confirming the presence of an undifferentiated neuroblastoma with varying degree of stroma and the presence of Homer‐Wright pseudorosettes characteristic of neuroblastoma***.*** In the s.c. model, all animals in the study reached the humane end point by day 35 (Fig. S1B). In the systemic model, all animals reached their humane endpoints by day 50 (Fig. S1D). Necropsy of the animals indicated that the tumor growth was largely confined to the livers which were enlarged (liver weights were three‐times that of control NRG mice) and infiltrated with metastatic nodules.

**Figure 5:**
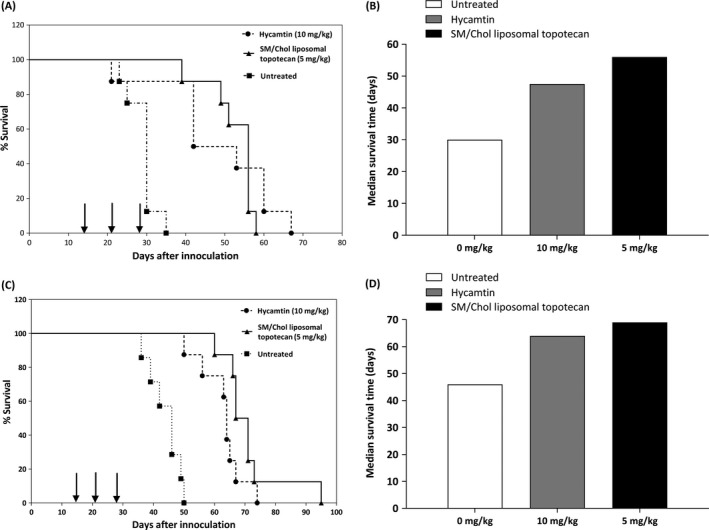
SM/Chol liposomal topotecan exhibits improved therapeutic activity compared to two‐times the dose of Hycamtin. The antitumor activity of topotecan was assessed against the s.c. (panels A and B) and systemic (panels C and D) LAN‐1 neuroblastoma models following administration of SM/Chol liposomal topotecan (5 mg/kg) or Hycamtin (10 mg/kg) where the drug was administered intravenously on day 14, 21 and 28 (arrows). Kaplan–Meier survival plots (A, C) and median survival times (B, D) are shown. Survival curves were determined based on when the mice reached a humane endpoint as defined in the Methods. The day of death was recorded 1 day following euthanasia. The efficacy studies were completed using groups of 8 mice per dose tested. Although an increase in mean survival time by using SM/Chol liposomal topotecan, the differences with Hycamtin treatment in both models were not statistically significant by the log‐rank test, *P *>* *0.05.

The antitumor activity of topotecan administered (i.v., Q7D × 3) as the SM/Chol liposomal formulation or Hycamtin was evaluated and the results, summarized in Figure 5, indicated in both models that the therapeutic activity of the SM/Chol liposomal topotecan was greater than Hycamtin when given at two‐times the dose. NRG mice with subcutaneous tumors treated with SM/Chol liposomal topotecan at 5 mg/kg exhibited a median survival time (MST) of 56 days compared to a MST of 47.5 days for Hycamtin administered at 10 mg/kg. For the NRG mice with the systemic neuroblastoma model, treatment with SM/Chol liposomal topotecan at 5 mg/kg increased the MST to 69 days, with some of the animals surviving up to 92 days. In contrast, when treated with Hycamtin at its maximum feasible dose (10 mg/kg) the MST was 64 days. While increases in therapeutic activity were observed in both subcutaneous and neuroblastoma models when SM/Chol liposomal topotecan was used relative to Hycamtin, the increases were modest and not statistically significant by the log‐rank test (*P *>* *0.05).

## Discussion

Neuroblastoma is the most common extracranial solid malignancy during early childhood 3. Topotecan (Hycamtin) is a drug that is currently being incorporated into initial induction chemotherapy, as well as first‐line salvage regimens, for this aggressive cancer 14, 16, 18, 34. Topotecan is a cell cycle specific agent and is most lethal during the S‐phase. For this reason, optimal antitumor activity is achieved when cancer cells are exposed to the active form of the drug (lactone form) for as long as possible 20, 21, 35. This effect is exemplified by the data summarized in Figure 1. Prolonged exposure time to the drug allows a greater proportion of cells to enter the S‐phase, consequently enhancing the therapeutic activity of the drug. Enhanced exposure times can be achieved using a number of approaches including continuous drug infusions 36, 37, more frequent dosing (i.e. metronomic dosing 38, 39, 40), PEGylation of camptothecin derivatives 41, 42, 43 and through use of nano‐scale drug delivery systems such as the liposomal formulations described here. Furthermore, topotecan's activity depends on maintaining an intact lactone ring and the liposomal formulations of topotecan will help maintain the drug in the lactone form for extended time periods following administration. This is due to the fact that in the plasma compartment the encapsulated drug is maintained in a low pH environment within the liposome.

The formulation modifications described in this report relied on the use of SM/Chol to engender further decreases in the rate of topotecan dissociation from the liposomes. SM has been used in previous liposomal anticancer drug formulations, in part because it is more stable than DSPC 44. SM lacks ester‐linked acyl chains that are present in DSPC and this property decreases SM susceptibility to hydrolysis or enzymatic degradation 44. In addition, it was shown to enhance drug retention in cholesterol containing liposomes and the decrease in membrane permeability may be due to the higher affinity between SM and cholesterol 31, 45. Here, the resulting SM/Chol formulation is less toxic than the previously described DSPC/Chol liposomal topotecan formulation (Topophore C^™^) developed in our lab and it also exhibited enhanced circulation longevity. The SM/Chol liposomal formulation increased topotecan plasma AUC_0–24 h_ by 1000‐fold when compared to the AUC_0–24 h_ of Hycamtin and by threefold when compared to the previously described Topophore C^™^ 27.

This is the first report assessing the therapeutic activity of liposomal topotecan in models of neuroblastoma. Activity was established in s.c. and systemic models of neuroblastoma established in NRG mice following inoculation of LAN‐1 cells. SM/Chol liposomal topotecan administered at 5 mg/kg extended the median survival time (MST) in comparison to Hycamtin given at 10 mg/kg. We attempted to relate the drug doses used in these studies to topotecan doses currently used in neuroblastoma patients. In an hypothetical advancement of the formulation to a phase I clinical trial, considering that 5 mg/kg liposomal topotecan is a close dose to the maximum tolerated dose in mice and taking the previously cited Km factors as reference 46, the starting dose in human (1/10 of maximum tolerated dose) would be calculated as approximately 1.5 mg/m^2^. This value is similar to the current dosages of topotecan which are given to patients with neuroblastoma in the clinics that is 0.75 mg/m^2^/day 34.

**Figure 6:**
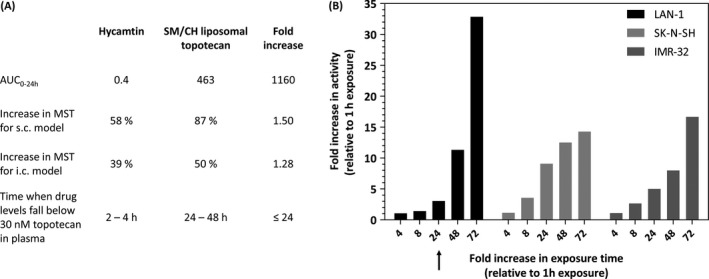
Relating why a substantial increase in AUC only results in a modest gain in therapeutic efficacy for liposomal topotecan. (A) The increase in AUC observed in this study was more than 1000‐fold when comparing free topotecan to the liposomal topotecan, hence it was disappointing that the activity in vivo was not enhanced substantially when comparing the liposomal drug to the free drug. This simplistic analysis, however, did not consider the effects of drug concentration. So while there was a great increase in AUC, the drug levels in the plasma compartment fell below the IC_50_ for LAN‐1 cells (30 nmol/L) at 2–4 h for the free drug and 24–48 h for the liposomal drug. When expressed this way, the increased time to which the tumor cells were exposed to drug levels greater than the IC_50_ was at most 24‐fold. (B) Based on this analysis, one would expect that the liposomal formulation would only be at most about threefold more active than the free drug, since a 24‐fold increase in drug exposure time in vitro only results in a threefold decrease in IC_50_ (see arrow). Theoretically, following this rationale, drug concentration in plasma should be sustained above 30 nmol/L for longer periods of time (for example 48 h or 72 h) to achieve higher increases in therapeutic efficacy. Data presented in panel A is from Figure 4A and 5; panel B is a reinterpretation from Figure 1C.

Although an improvement in efficacy by the liposomal delivery of topotecan, the magnitude of the effect was not as great as expected from the changes in topotecan exposure achieved in the plasma compartment and tumor. This may be due to the possibility that the extension in exposure time of drug to tumor cells was still not sufficient to demonstrate a remarkable improvement in activity (Fig. 6). Tumor cells were only exposed 24‐folds longer to topotecan when the drug was administered as liposomal formulation (Fig. 6A) but the cytotoxicity just increases substantially after a 48‐fold increase in exposure time (Fig. 6B). Therefore, the retention time of the liposomal formulation may still be too short and it should be further extended to at least 48 h, so that a more pronounced increase in therapeutic efficacy is achieved. Another contributing factor could be that some of the measured topotecan is still sequestered in the liposomes and not available to the cancer cells. In any case, we conclude that further optimization of the formulation's ability to retain topotecan still needs to be made in order to achieve meaningful improvements in efficacy.

We will focus our future research on developing the therapeutic potential of this topotecan formulation through its use in combination with other agents used in the management of neuroblastoma such as anti‐GD2 antibodies or radiopharmaceuticals. The novel SM/Chol topotecan formulation has considerable pharmaceutical potential, and future studies will assess its activity in combination with ^131^I‐MIBG 14, 47 and Dinutuximab 48; studies that will evaluate whether these targeted therapeutic agents exhibit synergistic anti‐tumor activity when associated with the optimized liposomal topotecan.

## Conflict of Interest

The authors declare that they have no conflict of interest with the financial institutions that supported this research.

## Supporting information


**Table S1.** Tolerability studies in NRG mice following administration of Hycamtin or SM/Chol liposomal topotecan (Q7D × 3).
**Figure S1.** LAN‐1 neuroblastoma model development studies. Hematoxylin and Eosin staining of tumors harvested after subcutaneous injection of LAN‐1 neuroblastoma cells (panel A) show Homer–Wright pseudorosettes (black arrow) characteristic of neuroblastoma. Kaplan–Meier survival plot for animals bearing subcutaneous LAN‐1 tumors, where the humane endpoint was defined by tumors exceeding 800 mm^3^ (panel B). Kaplan–Meier survival plot for animals given intracardiac (i.c.) injections of LAN‐1 cells, where the humane endpoints were defined by body condition score, weight loss and behavioral changes (panel D). Animals that succumbed to tumor progression following i.c. injection of LAN‐1 cells exhibited large livers with numerous associated tumors. A Hematoxylin and Eosin stain section of liver associated tumors is provided in panel C.
