# Supplementary material for: Optimization of liposomal topotecan for use in treating neuroblastoma
Source: Cancer Med. 2017 May 23;6(6):1240–54. doi: 10.1002/cam4.1083 (PMC5463073; doi:10.1002/cam4.1083)
Supplement: Supplementary file 1 — Table S1. Tolerability studies in NRG mice following administration of Hycamtin or SM/Chol liposomal topotecan (Q7D × 3). Figure S1. LAN‐1 neuroblastoma model development studies. Hematoxylin and Eosin staining of tumors harvested after subcutaneous injection of LAN‐1 neuroblastoma cells (panel A) show Homer–Wright pseudorosettes (black arrow) characteristic of neuroblastoma. Kaplan–Meier survival plot for animals bearing subcutaneous LAN‐1 tumors, where the humane endpoint was defined by tumors exceeding 800 mm3 (panel B). Kaplan–Meier survival plot for animals given intracardiac (i.c.) injections of LAN‐1 cells, where the humane endpoints were defined by body condition score, weight loss and behavioral changes (panel D). Animals that succumbed to tumor progression following i.c. injection of LAN‐1 cells exhibited large livers with numerous associated tumors. A Hematoxylin and Eosin stain section of liver associated tumors is provided in panel C. [file CAM4-6-1240-s001.doc]

**Supplementary Information**

**Table S1:** Tolerability studies in NRG mice following administration of Hycamtin or SM/Chol liposomal topotecan (Q7D x 3).

| **Schedule** | **Formulation** | **Dose (mg/kg)** | **Major signs of toxicity**^(3)^ |
| --- | --- | --- | --- |
| **(Q7D x 3)** | **SM/Chol liposomal topotecan**^(1)^ | 2.5 | No weight loss |
|  |  | 5 | No weight loss |
|  |  | 7.5 | 1-5% weight loss (4/4) |
|  |  | 10 | 5-12% weight loss (2/4), sunken eyes, scruffy, skin tented, diarrhea (1/4) |
|  | **Hycamtin** | 10^(2)^ | 1-5% weight loss (1/4),hyperactive, sunken eyes, scruffy, skin tented, hunched, diarrhea, loss of righting reflex (1/4). |

^(1)^ Prepared at 0.025 drug-to-lipid ratio (mol: mol), particle size ~100nm.

^(2)^ 10mg/kg was the maximum feasible dose (MFD) of the clinical product available.

^(3)^ Established using a Standard Operating Procedure designed to assess signs of toxicity. The staff recording the signs of toxicity were blinded to the treatment groups.


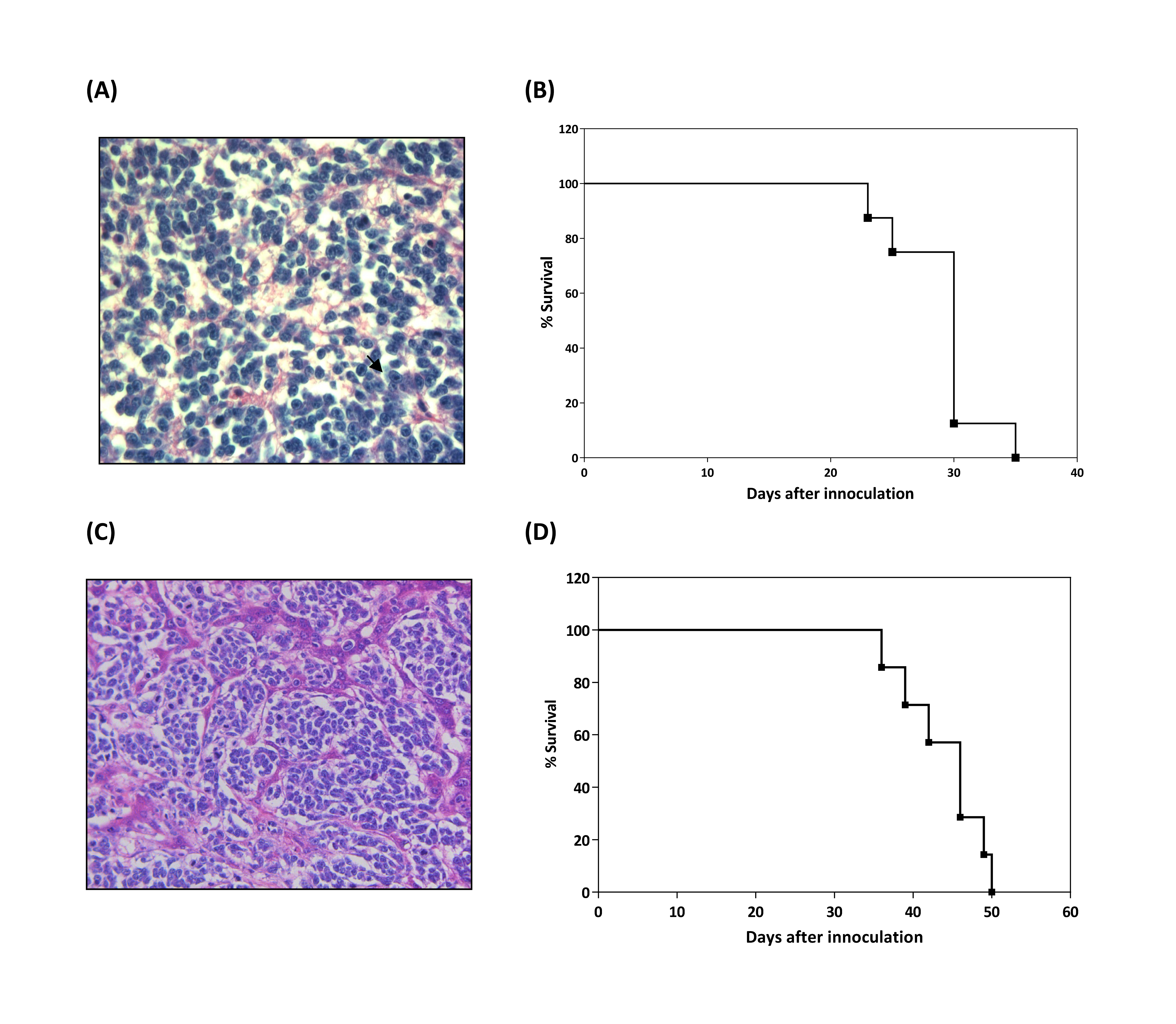


**Figure S1:** LAN-1 neuroblastoma model development studies. Hematoxylin and Eosin staining of tumours harvested after subcutaneous injection of LAN-1 neuroblastoma cells (panel A) show Homer-Wright pseudorosettes (black arrow) characteristic of neuroblastoma. Kaplan-Meier survival plot for animals bearing subcutaneous LAN-1 tumours, where the humane endpoint was defined by tumours exceeding 800mg (panel B). Kaplan-Meier survival plot for animals given intra-cardiac (i.c.) injections of LAN-1 cells, where the humane endpoints were defined by body condition score, weight loss and behavioural changes (panel D). Animals that succumbed to tumour progression following i.c. injection of LAN-1 cells exhibited large livers with numerous associated tumours. A hematoxylin and eosin stain section of liver associated tumours is provided in panel C.
